# The Effect of Conduction Exercise and Self-Acupressure in Treatment of Parkinson's Disease: A Pilot Study

**DOI:** 10.1155/2020/7950131

**Published:** 2020-08-11

**Authors:** Chun-Sum Yuen, Ka-Kit Chua, Wai-Hing Lau, Zhi-Yuen Zhuang, Ho-Yan Chow, Min Li

**Affiliations:** ^1^School of Chinese Medicine, Hong Kong Baptist University, Kowloon Tong, Hong Kong; ^2^Mr. & Mrs. Ko Chi-Ming Centre for Parkinson's Disease Research, Hong Kong Baptist University, Kowloon Tong, Hong Kong

## Abstract

**Introduction:**

Parkinson's disease cannot be well treated by conventional medication. Acupuncture and Tai Chi are proven to be effective in relieving symptoms of Parkinson's disease. Traditional Chinese medicine exercises may prove to be an effective complementary therapy.

**Objective:**

To evaluate the efficacy and safety of conduction exercise and self-acupressure in treating Parkinson's disease.

**Method:**

This study is an accessor- and data analyzer-blind, add-on, randomized, controlled, pilot clinical study. In the treatment group, they were taught to practice conduction exercise and self-acupressure for 8 weeks. No additional treatment was given in the control group. Assessments were done at week 4 and week 8 of the treatment period. The primary outcomes are the total score and domain scores of the Chinese version of 39-item Parkinson's Disease Questionnaire. The secondary outcomes are the total score and domain scores of a custom-designed questionnaire, which is a short form of Nonmotor Symptom Scale.

**Results:**

22 patients in the treatment group and 14 in the control group continued to the treatment phase. Patients in the treatment group displayed improvement trends in primary and secondary outcomes. Improvements were significant in two areas of a custom-designed questionnaire: total score (*p*=0.014) and domain score of gastrointestinal tract (*p*=0.004). No severe adverse events were reported.

**Conclusion:**

Conduction exercise and self-acupressure were well accepted by and feasible for Parkinson's disease patients. The data generated can be used for the planning of future studies. The exercise regime can be promoted as a home-based, self-practice therapy for Parkinson's disease patients, due to its safety, low cost, and convenience in implementation. This study is registered with the Chinese Clinical Trial Registry (ChiCTR-IPR-17011987, on 14 July 2017).

## 1. Introduction

Parkinson's disease (PD) is the second most frequently observed neurological disorder in the world. According to an epidemiological study, it has been found that around 13,000 people are suffering from PD in Hong Kong, with the prevalence in the age group of ≧55 years to be approximately 0.5% [1]. The etiology of the disease is unknown, but the main pathological changes of PD are the loss of nigrostriatal dopaminergic neurons in the substantia nigra pars compacta (SNpc) and aggregation of Lewy body [2, 3].

The four cardinal symptoms of PD, which are motor symptoms (MS), are tremor, bradykinesia, postural instability, and rigidity, while nonmotor symptoms (NMS) include constipation, muscular pain, and fatigue [4]. NMS is reported to be a key determinant for the health-related quality of life (HR-QoL) and causes great distress to PD patients [5, 6].

Levodopa is currently the most potent drug against PD [7]. Long-term use of levodopa, however, inflicts motor-related complications such as motor fluctuations and dyskinesia. These complications are reported to have a large impact on the HR-QoL of PD patients [8]. Since current medications bring about adverse effects on patients, many alternative therapies and nonpharmacological treatments are being explored.

The disease in Traditional Chinese medicine (TCM) that largely encompass PD is “Chan Zheng,” or Tremor Syndrome [9]. CZ is caused by a combination of pathological waste such as static blood and phlegm and deficiency in kidney, liver, and spleen. These causes lead to insufficient nourishment to sinews and meridians in the extremities [10]. Our previous research also discovered that a large portion of PD patients had “Deficiency of Spleen Qi” (DSQ) [11]. The treatment strategy will be to reinvigorate internal organs and nourish meridians [12].

Exercises have been found to improve the HR-QoL of PD patients [13]. Tai Chi, for one, is reported to be effective in improving the motor function of PD patients, as well as enhancing the circulation of meridians and reinvigorating internal organs [14, 15]. Acupuncture is also efficient in improving the condition of PD and boosting the effect of conventional medications [16, 17]. Based on this evidence, a TCM exercise regime, combining conduction exercise (CE) and self-acupressure (SA), was designed for PD patients, and its effectiveness is tested in this clinical trial [18].

CE is a set of maneuvers combining rhythmic breathing and bodily movements [19]. The aim is to attain peace of mind and relaxation in the body [20]. The CE used in this study was named “Nine Rotations of Longevity” (NRL) [21]. The exercise focused primarily on self-massaging the abdominal area to reinvigorate expulsion of pathological products, nourish internal organs, defense against harmful substances, and restore the balance of Yin and Yang. The premise was that NRL could improve NMS of PD, such as constipation, fatigue, and insomnia. Acupressure, often practiced with CE, is the massage of acupoints [22]. If it is done by one's self, it is called self-acupressure (SA). The SA used in this study focuses on relieving MS such as decreased muscle strength, myalgia, and rigidity [23, 24].

The literature review done before the commencement of the study concluded that no prior clinical research studied CE and SA in treating PD. Therefore, a pilot study is designed to provide evidence that SA and CE are viable alternatives, or complementary, therapies in treating PD. The study is a randomized, assessor- and data analyzer-blind, controlled add-on trial with restrictive inclusion and exclusion criteria.

This study hypothesizes that CE and SA could diminish pathological products and replenish organ deficiencies caused by PD, thus allowing meridians to be unblocked and the harmonious balance of internal organs restored. After practicing CE and SA, we expect that MS and NMS could be improved.

## 2. Methods

### 2.1. Study Objective

To evaluate the efficacy and safety of the CE and SA exercise in treating both motor and nonmotor symptoms of PD.

### 2.2. Trial Design

As reported in our study protocol, this study is an 8-week, assessor- and data analyzer-blind, add-on, randomized, pilot clinical trial [25]. It is approved by the Ethics Committee of the Hong Kong Baptist University's (HKBU) Institutional Review Board (code: HASC/16-17/630) and registered with the Chinese Clinical Trial Registry (ChiCTR-IPR-17011987, registered on 14/7/2017). Patients with mild to moderate PD were recruited through Hong Kong Parkinson's Disease Association. Eligibility screening was conducted through the phone. After baseline assessment, eligible patients were randomly placed, in a 1 : 1 ratio, into treatment group or control group. Patients in the treatment group were to take part in an 8-week practice of CE and SA. The control group received no additional intervention, aside from their usual treatment. Assessments were conducted at week 4 (midpoint) and week 8 (endpoint). The site of study and data collection was at the School of Chinese Medicine in HKBU. Before the commencement of treatment period, participants signed an informed consent form explaining the details and their involvement in this study (Supplementary Material 1). A recent study revealed that clinical studies of Chinese medicine were not methodologically sound when evaluated by Consolidated Standards of Reporting Trials (CONSORT) statement [26, 27] (Supplementary Material 2). In a hope that future clinical studies of Chinese Medicine will be done with a higher quality, our clinical trial was done with rigor, conforming to the guidelines of CONSORT.

### 2.3. Participants

Eligible participants of this study fulfilled the following inclusion criteria: (1) clinical diagnosis of PD based on the criteria of the United Kingdom Parkinson's Disease Society Brain Bank Clinical Diagnostic Criteria (UKPDBB) [28]; (2) age between 18 and 80 years; and (3) treatment with levodopa, alone or in combination with other antiparkinsonian drugs, stable over the previous two months.

The exclusion criteria of this study were as follows: (1) atypical or secondary parkinsonism; (2) use of antidepressants during the preceding month; (3) history of psychosis; (4) suicide attempts during the preceding 12 months; (5) severe diseases (i.e., cancer, stroke, and acute present heart attack); (6) participating in other behavioral or pharmacological studies; (7) pregnancy or breastfeeding; (8) surgery within preceding two months or having a scheduled operation within the period of study; or (9) Hoehn and Yahr (H&Y) stage ≧4 [29], with activities limited to wheelchairs.

### 2.4. Patient Involvement

We encouraged participants to give feedback regarding the conduct of the study and the design of intervention after the commencement of the study. The opinions from family members were also sought, as they were the major caregivers of patients. Improvements were made so that the study can be more patient-friendly.

### 2.5. Study Intervention

For patients in the treatment group, they were instructed to practice an exercise regime combining CE and SA. The regime was designed by our research team and was primarily taught by research assistant I (RA-I), Yuen.

During the treatment period, the exercises were then taught in 8 weekly sessions, each 1 to 1.5 hours long. Individual performances of the exercise were evaluated by the instructor, RA-I, to ensure overall intervention quality, starting from week 4. Extra time was given to those who failed to meet the standard. Aside from the weekly sessions, patients were instructed to practise every day at least once and twice at maximum. Additionally, they were required to not make changes to their previous treatments.

Aids were given to patients to facilitate practice at home. A booklet containing all 14 steps of CE and SA with detailed depiction and pictures was given to patients (included in Supplementary Material 3). An individual journal for every patient was also kept for the record of the number of times of practice at home. For more information on the design of CE and SA, refer to Supplementary Material 3.

As for participants in the control group, they received two sessions of health-related talk which have no direct therapeutic effect.

### 2.6. Randomization and Masking

For allocation of groups, patients were placed in treatment or control group by a ratio of 1 : 1 through randomization. Excel was used to generate the randomization number sequence. Research assistant II (RA-II) Zhuang was responsible for monitoring the entire randomization process. All related materials were secured by a password in a computer manned by RA-II. A sealed opaque envelop was used to store the hardcopy of the allocation list. The assessors and the data analyzer were not made aware of the allocation and granted no access to the related material during the study. RA-II had no direct contact with participants, nor did he participate in recruitment and screening.

### 2.7. Sample Size Calculation

For the calculation of sample size of pilot studies, it can be derived using “the rule of 12”, a finding concluded by a recent paper on sample size estimation [30]. The rule stated that a single treatment arm should consist of 12 or more participants in order to obtain a reliable effect size estimation.

### 2.8. Outcome Measurements

The primary outcomes for this study were the total score and domain total scores of the Chinese version of 39-item Parkinson's Disease Questionnaire (C-PDQ-39) [31]. C-PDQ-39 is a study-validated and universally used assessment for PD patients. The questionnaire consists of 39 questions evaluating MS, NMS, and HR-QoL. The domains are mobility, activities of daily living, emotional well-being, stigma, social support, cognition, communication, and bodily discomfort. As for the secondary outcomes, 4 domains of Nonmotor Symptom Scale (NMSS), a research-validated assessment evaluating NMS, were extracted and used as a custom-designed questionnaire (CDQ) [32] (Supplementary Material 4). The 4 domains are sleep/fatigue, gastrointestinal tract, urinary, and miscellaneous. We initially planned in the protocol to include DSQ scoring in CDQ. However, considering the internal consistency of the questionnaire, the extensiveness of NMSS in evaluating NMS, and Chinese medicine symptoms, we believed that the sole usage of NMSS would suffice. To measure the internal consistency of CDQ, Cronbach's alpha reliability coefficient was used. The resulting coefficients of the overall score and the domain scores of CDQ were all larger than 0.7, which is deemed as “acceptable” [33, 34]. For both primary and secondary outcomes, the higher the scores, the more severe the symptoms and conditions.

Co-investigator (Co-I) K.K. Chua supervised all outcome measurements. Research assistant III (RA-III) H.Y. Chow was responsible for conducting outcome measurements. Chua is a registered Chinese medicine practitioner and was the person in charge of training all assessors in this study. Both were not aware of the allocation of participants. Patients were instructed to take their antiparkinsonian drugs before outcome assessments, so that they were at “on” state in all assessment sessions.

### 2.9. Data Management

All soft copies of data were secured with a password. All data in hardcopy were stored in a lock safe. Data analysis was conducted when treatment phase was completed. 3 years after end of research, all documents and data will be destroyed. Input and analysis of data were done by research assistant IV (RA-IV), W.H. Lau, with no access granted to the other investigators. RA-IV had no direct contact with any of the participants.

### 2.10. Statistical Analysis

In the protocol, it was planned that the change-score method and Mann–Whitney *U* test would be used to analyze study outcomes due to the small sample size. However, to provide a more accurate estimation of effect, analysis of covariance (ANCOVA) was used instead as it provides statistical control over confounding variables as covariates. It is also a robust test that does not require strictly a normal distribution of data. Therefore, the comparison of outcomes at time points adjusted for baseline assessments between the treatment group and the control group was measured using ANCOVA. For baseline characteristics, continuous data, such as dosage of medication and age, were compared using *t*-tests. Categorical data, such as gender distribution, were compared by the chi-square test. Our study implemented the intention-to-treat (ITT) principle, so that the data of all those who completed baseline assessment were included in data analysis. Last-observation-carried-forward (LOCF) principle was used to manage missing data. Analyses were carried out with SPSS 24.0 package (SPSS, Chicago, IL). No interim analyses were conducted.

## 3. Results

### 3.1. Recruitment and Enrollment

**Figure 1:**
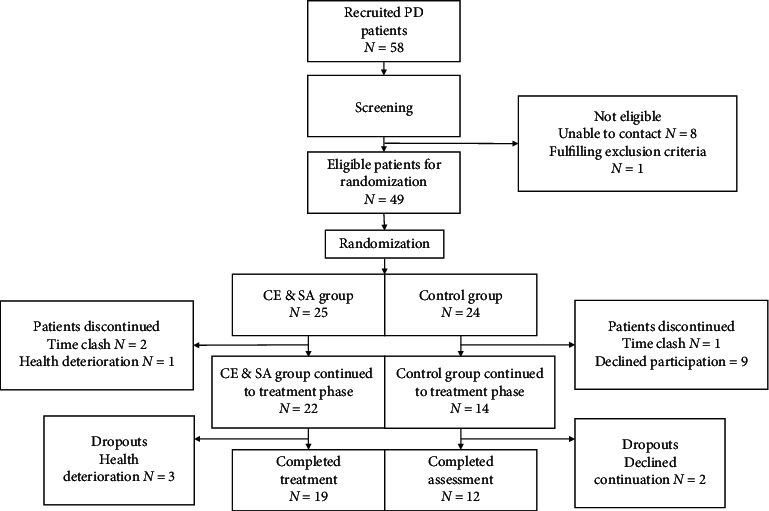
Flowchart of patient enrollment and participation statistics.

The recruitment period of the study was from June 2017 to July 2017. A total of 58 patients were recruited, of which 49 patients were eligible after screening (Figure 1). 9 patients were screened out due to failure to establish contact or fulfilling the exclusion criteria. After randomization, patients were allocated to the two groups in a 1 : 1 ratio, with 25 patients in the CE and SA group and 24 in the control group. There were dropouts after the allocation of groups and before the commencement of treatment, i.e., during the 1-week run-in period. In the CE and SA group, 2 patients withdrew due to time clash and 1 due to health deterioration, whereas, in the control group, 9 patients discontinued due to unwillingness and 1 due to time clash. By the initiation of the treatment phase, 22 patients were in the CE and SA group (9 males and 13 females; median age: 60.00 ± 9.00), and 14 were in the control group (4 males and 10 females; median age: 65 ± 13.25).

At the end, 19 patients in the CE and SA group completed the intervention and all assessments, while 12 in the control group completed all assessments. A comparison of baseline demographics concluded that there is no statistically significant difference between the treatment and control groups (Table 1).

### 3.2. Acceptability and Feasibility

Patients in the CE and SA group attended at least 7 out of 8 weekly sessions. The journals recording the frequency of practice at home revealed that all patients practiced CE and SA once in 80% of days throughout the 8-week treatment phase. The suggestions of intervention improvement by patients were as follows: more practice time during weekly sessions, recording a video guide for practicing the exercise regime, and reducing the intensity of the exercise.

### 3.3. Results of Primary Outcome

With reference to Table 2, patients in the CE and SA group displayed improvement trend in areas including total score and domain scores of emotional well-being, stigma, social support, cognition, and bodily discomfort. ANCOVA presented negative adjusted mean difference compared with the control group in these areas except emotional well-being and bodily discomfort (total score: mean difference: −2.25, SEM: 4.77, and 95% confidence interval (CI): −11.94 to 7.45; mobility: mean difference: −0.60, SEM: 2.06, and CI: −4.78 to 3.58; stigma: mean difference: −0.52, SEM: 0.65, and CI: −1.84 to 0.79; social support: mean difference: −0.35, SEM: 0.434, and CI: −1.23 to 0.54; cognition: mean: −0.41, SEM: 0.64, and CI: −1.70 to 0.89). However, the differences were insignificant (*p* > 0.05).

### 3.4. Results of Secondary Outcome

As shown in Table 3, there were negative adjusted mean differences compared with the control group in total score (mean difference: −11.24, SE: 4.32, and CI: −20.02 to −2.46) and domain score of gastrointestinal tract (mean difference: −5.24, SE: 1.71, and CI: −8.72 to −1.75), and the differences were significant (total score: *p*=0.014; gastrointestinal tract: *p*=0.004). Other domains did not display significant improvements (*p* > 0.05).

### 3.5. Adverse Events

Severe adverse events were not reported during the study (Table 4). There were 5 cases of adverse events declared in total (13.89% among all patients), with 4 being in the treatment group (18.18%) and 1 in the control group (7.14%). A case of mild leg bruising was reported (4.54%) in the treatment group, after a practice session of CE and SA. The other 4 cases had no direct correlation with the study.

## 4. Discussion

The primary hypothesis of CE and SA being able to bring about significant improvement to the primary outcome could not be supported by evidence. However, there was a significant improvement in the total score of CDQ, as well as in the domain score of gastrointestinal tract, showing that CE and SA are effective in improving symptoms of dribbling saliva, difficulty in swallowing, and constipation. CE and SA are deemed to be safe, acceptable, and feasible due to the absence of severe adverse events and high treatment compliance. As this is the first rigorous study of the TCM exercise regime on PD, related large-scale studies can be planned based on the results generated.

The results of our study suffered from discrepancies due to the relatively small sample size and a considerable rate of dropout. Although, as a pilot study, a sample size of 12 or above per arm is acceptable, studies have suggested that low sample size is more likely to produce unreliable results [35]. It is, therefore, necessary to conduct a large-scale study to validate and dig deeper into the effect of CE and SA.

One of the major reasons for a small sample size is due to dropouts throughout our study. Control group suffered the highest number of dropouts (10 prior to the start of the treatment phase and 2 during the treatment phase), with the reason being their dissatisfaction with the grouping result. It was suggested that in the future, CE and SA classes can be given to participants in the control group upon the completion of the treatment phase. It can serve as an incentive for them to continue their participation in the study. Another way to encourage participation is to host health talks with topics more related to PD patients. It was found that PD patients were expectant to learn TCM methods of treating PD, including diet therapy and breathing exercises (Qi Gong). Talks of related topics can be used in the future.

Another limitation of this study is that the outcome measurements, both primary and secondary, are solely based on patients' self-reported symptoms. As PD is a cluster of various symptoms, the presence of other problems may overshadow improvements. The mood is also an essential factor in affecting the assessment of self-reported symptoms. PD patients are subject to mood swings due to their fluctuating conditions, causing bias in outcome assessment. Inclusion of an objective test evaluating MS as outcome measurement should be included in future studies. Examples are “timed up and go test” [36], “six-minute walk test” [37], and “10-meter walk test” [38].

For this study, no sham treatment was introduced as control. As a TCM exercise treatment study, with the exercise regime being used for the first time in a clinical trial, there were difficulties in devising a sham treatment with subtherapeutic effects [39]. It was decided that a control group with no treatment would be more viable.

A large-scale study with a more extensive outcome and a cost-effectiveness analysis should be conducted to further investigate the effectiveness and economic benefits of CE and SA. Modification to the design of exercise regime should be made in future studies to overcome the limitations discovered. By employing CE and SA as an alternative therapy, it is hoped that PD patients' NMS can be relived, and their quality of life can be improved [40]. It is suggested that the exercise regime can be promoted as a home-based, self-practice therapy, due to its low cost and relative safety.

## 5. Conclusion

The findings regarding the effectiveness of primary outcomes were inconclusive. Nonetheless, CE and SA were found to be effective in treating NMS related to the gastrointestinal tract. The exercise regime was proven to be acceptable, feasible, and safe. It is, therefore, suggested CE and SA to be a possible alternative treatment for PD, especially for treating NMS. Further studies with a larger sample size and more precise outcome measurements are encouraged to be conducted to explore further the effectiveness of CE and SA.

## Figures and Tables

**Table 1:** Baseline demographics.

	CE and SA group (*N* = 22)	Control group (*N* = 14)	*p* value^*∗*^
Age (years)	63.77 ± 1.41	64.64 ± 2.57	0.75
Gender (M/F)^†^	9/13	4/10	0.45
Disease duration (years)	8.41 ± 1.27	8.64 ± 1.44	0.91
Duration of levodopa treatment (years)	5.47 ± 1.35	6.36 ± 1.47	0.67
Daily levodopa equivalent dose (mg)	491.77 ± 65.36	541.86 ± 86.31	0.64
Other antiparkinsonian drugs^†^			
Dopamine agonist, *n* (%)	12 (54.5)	7 (50.0)	0.79
Muscarinic antagonist, *n* (%)	7 (0.32)	3 (21.4)	0.71
COMT inhibitor, *n* (%)	1 (4.5)	1 (7.1)	1.00
MAO-B inhibitor, *n* (%)	9 (40.9)	5 (35.7)	0.76
Clonazepam, *n* (%)	3 (13.6)	1 (7.1)	1.00
Amantadine, *n* (%)	0 (0)	3 (21.4)	0.051

Data are expressed in the format of mean ± SEM; ^*∗*^*p* value was used to indicate the difference between the two groups in baseline, calculated with independent *t*-test; ^†^the *p* value was calculated using chi-square test or Fisher's exact test.

**Table 2:** Primary outcomes across study time points.

	CE and SA group (*N* = 22)	Control group (*N* = 14)	Between group	
	Mean ± SE	Mean ± SE	Adjusted mean difference ± SE^*∗*^ (95% CI)	*p* value^†^
Primary outcome C-PDQ-39				
Total				
Baseline	43.32 ± 4.75	40.64 ± 5.31		
Week 4	43.68 ± 4.76	42.79 ± 6.13	−1.50 ± 4.12 (−9.89 to 6.89)	0.72
Week 8	41.32 ± 5.22	41.07 ± 6.33	−2.25 ± 4.77 (−11.94 to 7.45)	0.64

Mobility				
Baseline	13.95 ± 1.62	16.57 ± 2.42		
Week 4	12.95 ± 1.65	15.90 ± 2.80	−0.26 ± 1.87 (−4.06 to 3.53)	0.89
Week 8	12.77 ± 1.77	15.57 ± 2.70	−0.60 ± 2.06 (−4.78 to 3.58)	0.77

Activities of daily living				
Baseline	6.14 ± 1.02	7.00 ± 1.38		
Week 4	6.36 ± 0.99	7.14 ± 1.55	−0.052 ± 1.03 (−2.15 to 2.05)	0.96
Week 8	6.86 ± 0.97	7.29 ± 1.59	0.23 ± 1.24 (−2.29 to 2.74)	0.86

Emotional well-being				
Baseline	7.23 ± 1.06	4.57 ± 1.28		
Week 4	7.18 ± 1.16	4.79 ± 1.28	0.012 1.00 (−2.02 to 2.05)	0.99
Week 8	6.82 ± 1.22	4.21 ± 1.15	0.44 ± 1.22 (−2.04 to 2.92)	0.72

Stigma				
Baseline	3.64 ± 0.75	2.43 ± 0.47		
Week 4	3.59 ± 0.63	3.14 ± 0.79	−0.46 ± 0.69 (−1.86 to 0.95)	0.51
Week 8	3.05 ± 0.66	2.64 ± 0.71	−0.52 ± 0.65 (−1.84 to 0.79)	0.43

Social support				
Baseline	1.68 ± 0.44	1.14 ± 0.72		
Week 4	1.95 ± 0.41	1.50 ± 0.59	0.23 ± 0.63 (−1.05 to 1.51)	0.72
Week 8	1.41 ± 0.34	1.36 ± 0.75	−0.35 ± 0.43 (−1.23 to 0.54)	0.43

Cognition				
Baseline	4.36 ± 0.62	4.00 ± 0.70		
Week 4	4.64 ± 0.69	4.21 ± 0.74	0.097 ± 0.61 (−1.15 to 1.35)	0.88
Week 8	4.00 ± 0.57	4.14 ± 0.77	−0.41 ± 0.64 (−1.70 to 0.89)	0.53

Communication				
Baseline	2.14 ± 0.48	2.14 ± 0.55		
Week 4	2.91 ± 0.55	2.71 ± 0.55	0.20 ± 0.56 (−0.94 to 1.34)	0.72
Week 8	2.41 ± 0.48	2.93 ± 0.78	−0.514 ± 0.535 (−1.60 to 0.57)	0.34

Bodily discomfort				
Baseline	4.18 ± 0.50	2.79 ± 0.58		
Week 4	4.09 ± 0.59	3.79 ± 0.80	−0.78 ± 0.83 (−2.47 to 0.91)	0.35
Week 8	4.00 ± 0.55	2.93 ± 0.69	0.22 ± 0.79 (−1.40 to 1.84)	0.78

Data are expressed in the format of mean ± SEM; ^*∗*^the mean difference was derived through adjusting for baseline assessments of treatment and control group; ^†^*p* value was derived by ANCOVA.

**Table 3:** Secondary outcomes across study time points.

	CE and SA group (*N* = 22)	Control group (*N* = 14)	Between group	
	Mean ± SE	Mean ± SE	Adjusted mean difference ± SE^*∗*^ (95% CI)	*p* value^†^
Secondary outcome CDQ				
Total				
Baseline				
Week 4	38.41 ± 5.43	27.00 ± 6.28		
Week 8	40.68 ± 5.67	38.17 ± 6.51	−4.24 ± 4.00 (−12.38 to 3.90)	0.30
Sleep/fatigue	33.45 ± 5.86	34.57 ± 5.49	−11.24 ± 4.32 (−20.02 to −2.46)	0.014
Baseline				
Week 4	14.09 ± 2.00	11.14 ± 3.20		
Week 8	14.14 ± 1.85	11.71 ± 3.26	0.039 ± 1.99 (−4.01 to 4.088)	0.99
Gastrointestinal tract	9.91 ± 1.82	11.79 ± 2.94	−3.85 ± 2.29 (−8.50 to 0.80)	0.10
Baseline				
Week 4	9.95 ± 1.77	4.71 ± 1.39		
Week 8	10.27 ± 2.17	6.64 ± 1.69	−1.66 ± 1.86 (−5.44 to 2.12)	0.38
Urinary	7.95 ± 1.92	8.21 ± 1.89	−5.24 ± 1.71 (−8.72 to −1.75)	0.004
Baseline				
Week 4	5.36 ± 1.34	6.00 ± 1.90		
Week 8	5.95 ± 1.69	7.71 ± 1.75	−1.14 ± 1.25 (−3.67 to 1.40)	0.37
Miscellaneous	4.77 ± 1.69	7.5 ± 1.69	−2.17 ± 1.38 (−4.98 to 0.65)	0.13
Baseline				
Week 4	9.00 ± 2.20	5.14 ± 2.09		
Week 8	10.32 ± 2.31	8.14 ± 2.09	−1.00 ± 2.11 (−5.29 to 3.29)	0.64
	10.73 ± 2.30	7.07 ± 1.65	0.505 ± 1.83 (−3.22 to 4.23)	0.79

Data are expressed in the format of mean ± SEM; ^*∗*^the mean difference was derived through adjusting for baseline assessments of treatment and control group; ^†^*p* value was derived by ANCOVA.

**Table 4:** Adverse events reported by patients by group.

Adverse events	Number of patients (%)	
	CE and SA (*N* = 22)	Control (*N* = 14)
Bruise on leg	1 (4.54)	0
Upper respiratory infection	1 (4.54)	1 (7.14)
Fall	1 (4.54)	0
Change of dosage of PD medication	1 (4.54)	0

## Data Availability

The raw dataset used to support the findings of this study is available upon request.

## Supplementary Materials

Supplementary Materials Supplementary Material 1: informed consent form Supplementary Material 2: CONSORT 2010 Checklist guide to practicing CE and SA (it depicts the full procedure of practicing the exercise regimen). Supplementary Material 3: guide to practicing CE and SA (it depicts the full procedure of practicing the exercise regimen). Supplementary Material 4: custom-designed questionnaire (it shows all the questions listed in this questionnaire, which is the secondary outcome).Thank you and sorry for the trouble.
