# Supplementary material for: The Effect of Conduction Exercise and Self-Acupressure in Treatment of Parkinson's Disease: A Pilot Study
Source: Evid Based Complement Alternat Med. 2020 Aug 11;2020:7950131. doi: 10.1155/2020/7950131 (PMC7439182; doi:10.1155/2020/7950131)
Supplement: Supplementary Materials — Supplementary Material 1: informed consent form Supplementary Material 2: CONSORT 2010 Checklist guide to practicing CE and SA (it depicts the full procedure of practicing the exercise regimen). Supplementary Material 3: guide to practicing CE and SA (it depicts the full procedure of practicing the exercise regimen). Supplementary Material 4: custom-designed questionnaire (it shows all the questions listed in this questionnaire, which is the secondary outcome).Thank you and sorry for the trouble. [file 7950131.f1.zip › Supp Material 1 - Informed consent form.pdf]

Additional file 3 – Informed consent form

香港浸會大學
知情同意書

導引按穴對緩減柏金遜症的先導性臨床研究

您將被邀請參與一項臨床研究，評估中醫導引治療柏金遜症運動性及非運動性症狀。在你決定參與前，請務必清楚了解本研究的目的和所涉及的事項，並請你仔細考慮後才決定是否願意參加本研究。

資料背景
柏金遜症乃目前一種常見的腦部神經系統退行性病變，常見症狀除了肌肉運動障礙、震顫等運動性症狀，非運動性症狀亦不容忽視。根據本研究小組早前進行的研究結果顯示，大部分柏金遜症病人有脾氣虛的症狀，如便秘、腹脹等。因此，本研究目的為以隨機對照研究，觀察中醫導引法緩解柏金遜症運動性及非運動性症狀的療效。

是次研究將以原發性柏金遜症的診斷標準、研究的納入標準及排除標準進行篩選，最終將會篩選出 60 位原發性柏金遜症患者進行為期兩個月 (共 8 週)的臨床試驗研究，其中包括 8 次的集體導引教授和練習，及 3 次的症狀評估，參加者亦需在家中每天進行導引練習。在研究期間，參與者需同時服用西藥，並按隨機獲分派到治療組的或對照組。治療組需於研究過程每週到訪浸會大學，進行每次約 1 小時的導引練習 (一共八次)，並在第 0、4 及 8 週進行症狀評估。對照組只需於研究過程的第 0、4 及 8 週到訪浸會大學進行症狀評估。

請注意，在研究期間，參與者不得參與其他臨床研究；如閣下為女性，期間亦不可懷孕。此外，參與者尚需填寫日記以記錄日常生活的狀態及練習導引的時數。本臨床研究以隨機、對照的方式進行。

風險評估
本研究的導引方法安全可靠，一般不會引致任何不良反應，但仍不能保證個別人士可能出現肢體或軀體酸軟疼痛、頭昏目眩、腹部不適等症狀。若有關情況發生，臨床試驗將會終止，而有關參與者將獲得相應治療或被轉介予其它治療。

緊急醫療措施
在一般的情況下，臨床研究並不會對參與者身體造成損害。但是，若參與者出現因研究而導致的身體嚴重不適，本臨床研究將承擔因治療此嚴重不適而花費的費用。如果參與者在研究過程中出現任何不適，請盡快通知研究人員。除了相關醫藥費用之外，其它開支將不獲補償。

研究效益
參與此項臨床研究是完全免費的。參與者有可能藉此研究而改善柏金遜症的症狀，然而此療效並不能被保證。本研究計劃將能協助找出最安全和有效的導引方法緩解柏金遜症症狀。故此，您的參與將有助於中醫柏金遜症的治療研究及發展。

私隱保障

在研究開始時，每位參與者將獲隨機分配一個號碼記錄他們的分組編排，有關資料將會分別存放在密封文件夾及加密的電腦檔案內。整個研究過程中除了研究人員外，其他人士皆不可接觸有關資料。所有問卷、病人日記及其他相關文件亦只限研究人員查閱。記錄病程進展的文件將會以代號標示病人的個人資料。所有參與本臨床研究者的姓名、身份等個人私隱資料均獲保密，並在研究結果發表三年後予以銷毀。

補償

所有參與者若完成研究，可獲少量現金作交通津貼。如果您在參與是次研究時發生其他疾病，本研究小組並不會作補償安排。但若您在參與是次研究時發生與導引按穴直接相關的損害，在有充分理據下您可提出索償。此外，若在本研究過程中您認為研究者沒有按照上述的協定進行研究，或您認為您的個人權利受到侵犯，可電郵致 hasc@hkbu.edu.hk 與大學的人體及動物研究委員會作出投訴。

聯絡資料

如果您在參與研究過程中有任何疑問，可與阮俊森先生聯絡。
地址：香港九龍塘香港浸會大學中醫藥學院
電話：94379458
電郵：bucmpd@hkbu.edu.hk

參與條款

您明白參與是次研究乃是出於自己的意願，您可以在研究過程中退出而無需作出賠償。您的個人資料將在您退出後予以銷毀。

同意聲明

本人已閱讀並明白以上條文，並有機會發問。本人願意及決定參與本研究，並會獲得本同意書的副本。

______________________	______________________	________________
參與者/代理人姓名	參與者/代理人簽署	日期
______________________	______________________	________________
見證人姓名	見證人簽署	日期
______________________	______________________	________________
研究員姓名	研究員簽署	日期

－知情同意書完－

Hong Kong Baptist University

Informed Consent Form

The effect of conduction exercise and self-acupressure in treatment of Parkinson's disease: a pilot study

You are invited to participate a clinical trial studying the effectiveness of conduction exercise (CE) and self-acupressure (SA) in treating motor and non-motor symptoms (MS; NMS) of Parkinson's Disease (PD). Before you decide to participate, please make sure that the objective and all relevant details of the study are fully understood.

Background

Currently, PD is currently one of the most common neurodegenerative disease. While MS, such as rigidity and tremor, are the cardinal symptoms, NMS are not to be neglected. According to study done by our research team, a majority of PD patients suffer from Deficiency of Spleen Qi, a condition of weakened digestive system, with symptoms such as constipation and abdominal bloating. On the other hand, CE and SA are exercises practiced by ancient Chinese to boost health. Therefore, the aim of this study will be to observe the effectiveness of CE and SA in treating PD.

Patient participating in this study will be screened according to the diagnostic criteria of primary PD, inclusion and exclusion criteria of the study. There will be 60 eligible patients, including 8 practice sessions and 3 assessments in an 8-week treatment period. It will be required for patients to also practice the exercise at home. During the study, participants will be asked to resume their usual PD treatment with no alterations, and allocated randomly to either treatment or control group. For treatment group, they will need to attend an hour-long weekly session of CE and SA practice in Hong Kong Baptist University. Assessments will be done on Week 0, 4 and 8. Participants in control group will only be required to do assessments.

Risk assessment

The intervention used in this study is reliable and safe, will not cause any adverse effect. However, there may be some who will suffer from cases of limb soreness, dizziness and abdominal ache. Should the condition proven to be severe, patients will cease to continue in the study, and will receive treatment.

Emergency measures

Under normal circumstances, the study will inflict no harm onto patients. If, however, participants experience severe adverse effect due to the intervention, our research team will be responsible for all medical expenses required. Participants with any illness developed should alert our research team as soon as possible. Medical fees unrelated to the study will not be compensated.

Research objective

There is no participation fee for this study. It is possible that PD symptoms of participants will be relieved after practicing CE and SA, but their efficacy is not guaranteed. The aim of this trial is to derive the safest and most effective CE and SA regime in treating PD. Therefore, your participation would further the development of Chinese medicine in treating PD.

Privacy policy

Upon the commencement of the research, each participant will receive a randomly generated number for group allocation. The list of number will be kept in a sealed envelope, and the soft copy will be secured in a file of a locked computer. No personnel aside from the researchers responsible will have access to the data. All personal information of participants, completed questionnaires and

other related materials will not be disclosed, and will be destroyed three years after publication of results.

Compensation

All participants who have completed the treatment, will receive a small amount of travel compensation. Our research team will not be responsible for health problems unrelated to the study. If you experience health problems due to the study intervention, please seek compensation with sufficient proof. Should you feel that your rights are violated, or that the above agreement are not adhered, please contact hasc@hkbu.edu.hk to file complain.

Contact information

Please contact Mr. Yuen for enquiries during the course of research
Address: School of Chinese Medicine, Hong Kong Baptist University
Phone No.: 94379458
Email: bucmpd@hkbu.edu.hk

Participation agreement

You understand participation of this study is entirely voluntary. Withdrawal during the study will not be penalized. Your personal information will be destroyed after withdrawal.

Statement of informed consent

I have read and understood the above agreement, and was given to chance to make enquiries. I am willing to participate in this research, and will receive a copy of this informed consent form.
