# Supplementary material for: The Effect of Conduction Exercise and Self-Acupressure in Treatment of Parkinson's Disease: A Pilot Study
Source: Evid Based Complement Alternat Med. 2020 Aug 11;2020:7950131. doi: 10.1155/2020/7950131 (PMC7439182; doi:10.1155/2020/7950131)

Step-by-step guide to practicing Conduction exercise and Self-acupressure

- **Step 1**

  Place the three middle fingers (index, middle and ring fingers) of both hands at the epigastric area, and massage in a circular motion clock-wisely 21 times.

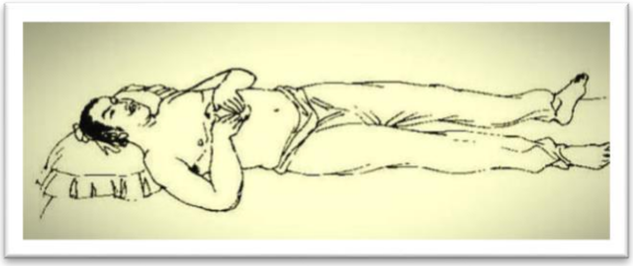

- **Step 2 & 3**

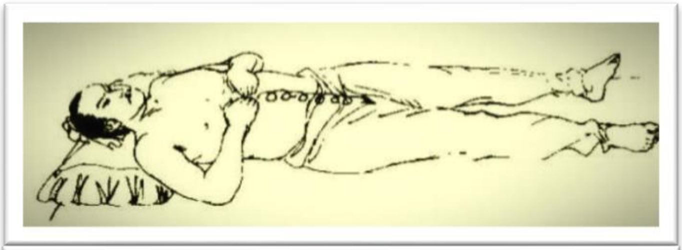

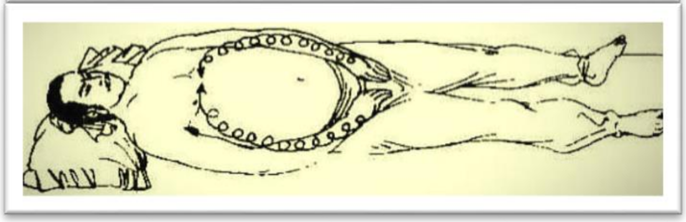

With the same fingers, massage gently in small circles from the epigastric area to the pubic symphysis under the belly button clock-wisely. With the same fingers, massage in small circles from the pubic symphysis area back to the epigastric area, with both hands driving separately from the two sides of the umbilicus,

Moves 2 and 3 should be done as a whole, and repeated 21 times as a set.

- **Step 4**

  With the same fingers, push in a straight line down towards the pubic symphysis. Repeat this action twenty-one times.

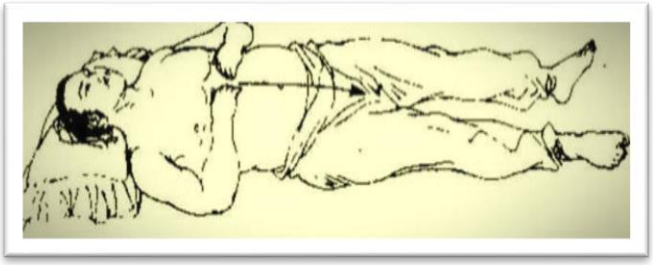

- **Step 5 & 6**

  Massage the umbilicus area from left to right in a clockwise manner with the right hand twenty-one times. Massage the umbilicus area from right to left in an anti-clockwise manner with the left hand twenty-one times

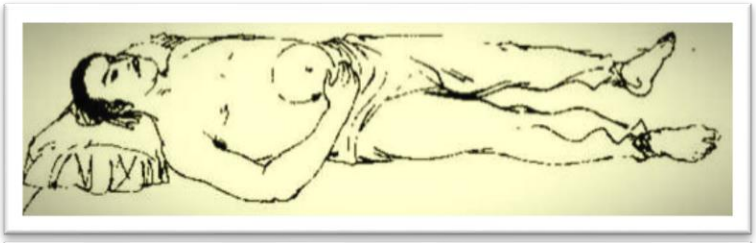

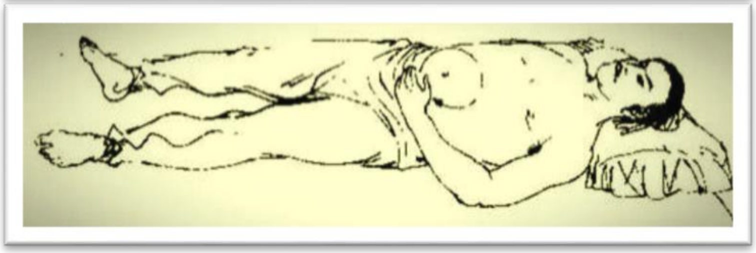

- **Step 7**

  Grasp the left hip area under the floating ribs with left hand, placing the thumb in the front and the other four fingers in the back; push straight down from the left chest to the groin using right hand twenty-one times。

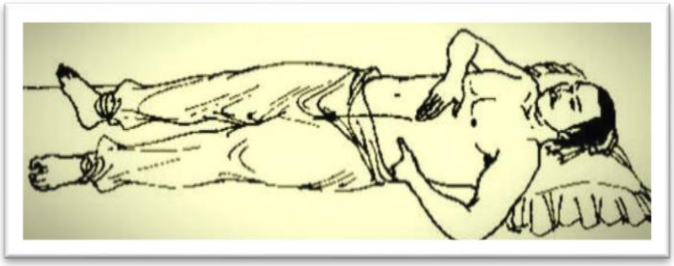

- **Step 8**

  Repeat the above action on the right side.

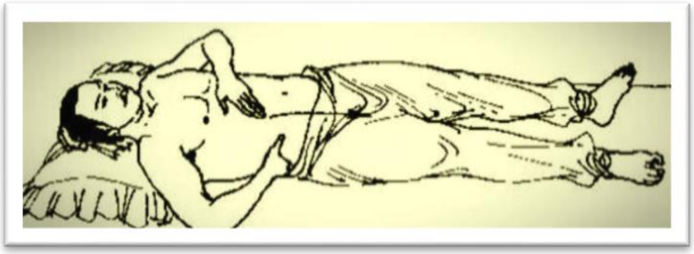

- **Step 9**

  In a seated meditative position, place two hands on the knees, and rotate the upper torso from left to right in an anti-clockwise manner 21 times, and from right to left in a clockwise manner, also 21 times.

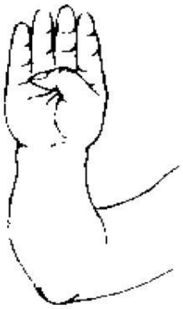

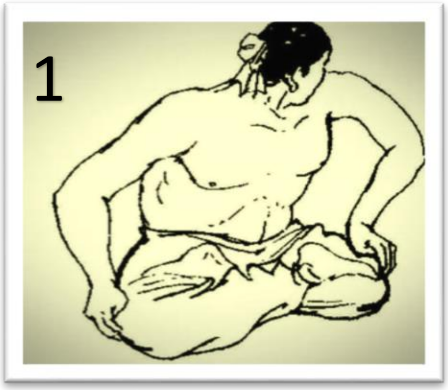

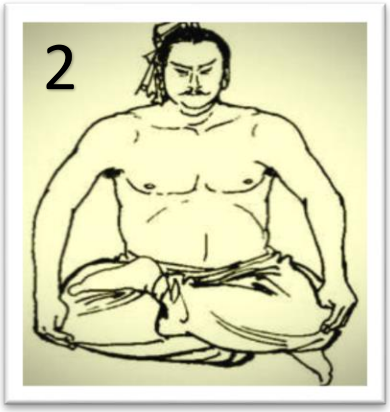

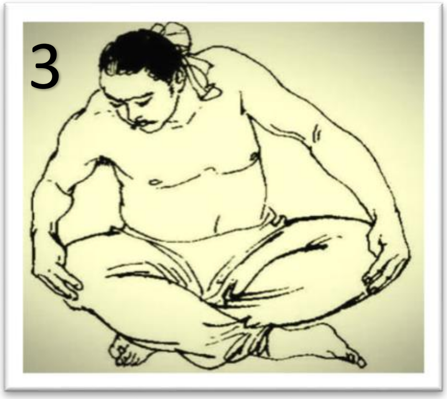

- **Points to note**:
  - Step 1 - 8 should be done in a supine position, while step 9 should be practiced in a seated position
  - While practicing, try to relax and be at peace

- **Step 10**

  With the fingers of both hands interlaced, place the palms of both hands at the back of the neck, and massage by compressing periodically 30 times in a minute.

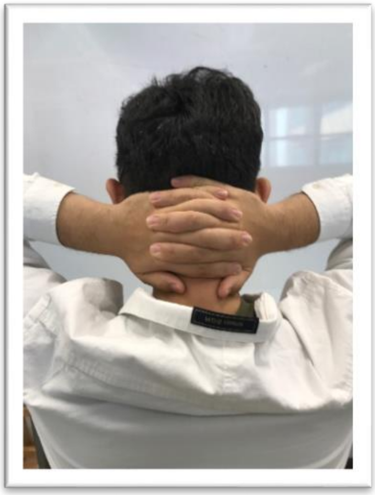

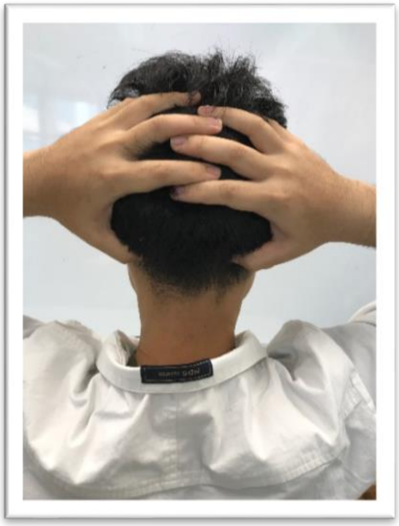

- **Step 11**

With the thumb, massage horizontally by pushing along the base of occipital bone from right to left 10 times in a minute

- **Step 12**

  Compress along the muscles and joints of the arm from shoulder to wrist, with four fingers (thumb excluded) on the lateral side and the palm on the medial side; compress 10 times in a minute for each arm

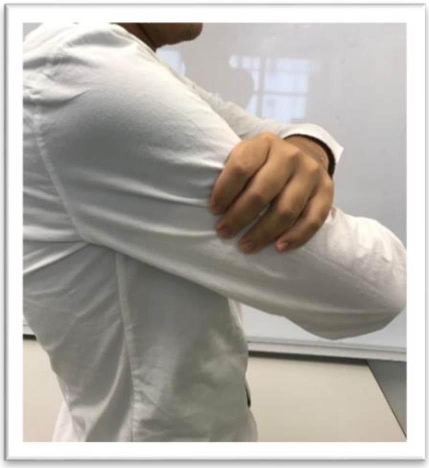

- **Step 13**

  Push firmly along the lateral and medial side of the thigh with the base of the palms of both hands; place the palms of both hands on both sides of the leg and compress from hip to ankle; complete the above set of actions 10 times in a minute for each leg

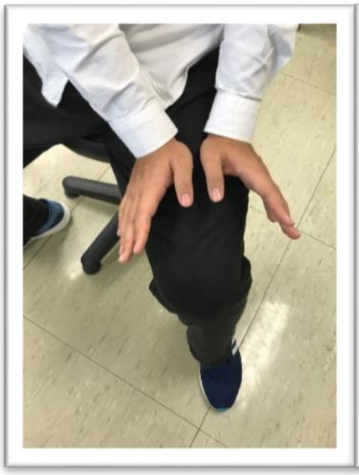
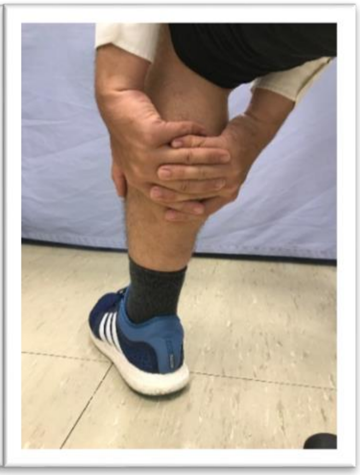

- **Step 14**

  Place both hands on the base of foot, and dorsiflex the both feet, and flex both knees and thighs to the maximum, and stay in this position for 10 seconds; relax both legs and plantar flex both feet to the maximum; complete the above set of actions 10 times

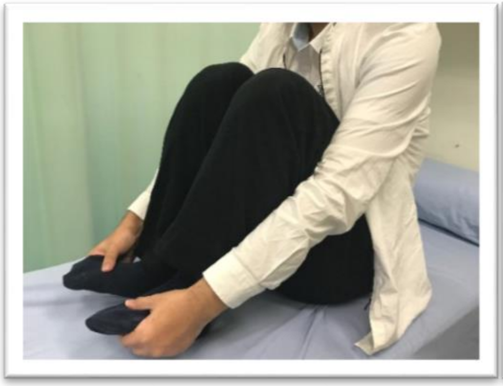
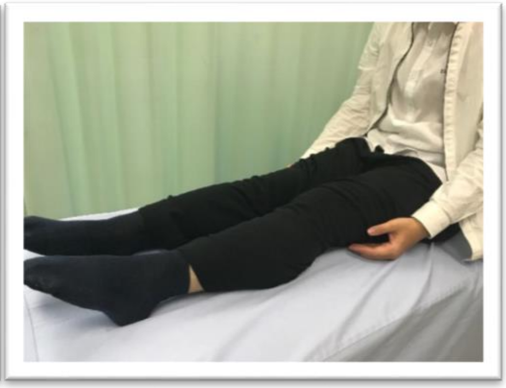

Supplement: Supplementary Materials — Supplementary Material 1: informed consent form Supplementary Material 2: CONSORT 2010 Checklist guide to practicing CE and SA (it depicts the full procedure of practicing the exercise regimen). Supplementary Material 3: guide to practicing CE and SA (it depicts the full procedure of practicing the exercise regimen). Supplementary Material 4: custom-designed questionnaire (it shows all the questions listed in this questionnaire, which is the secondary outcome).Thank you and sorry for the trouble. [file 7950131.f1.zip › Supp Material 3- Guide to practicing CE and SA.pdf]
