# Supplementary material for: The Effect of Conduction Exercise and Self-Acupressure in Treatment of Parkinson's Disease: A Pilot Study
Source: Evid Based Complement Alternat Med. 2020 Aug 11;2020:7950131. doi: 10.1155/2020/7950131 (PMC7439182; doi:10.1155/2020/7950131)
Supplement: Supplementary Materials — Supplementary Material 1: informed consent form Supplementary Material 2: CONSORT 2010 Checklist guide to practicing CE and SA (it depicts the full procedure of practicing the exercise regimen). Supplementary Material 3: guide to practicing CE and SA (it depicts the full procedure of practicing the exercise regimen). Supplementary Material 4: custom-designed questionnaire (it shows all the questions listed in this questionnaire, which is the secondary outcome).Thank you and sorry for the trouble. [file 7950131.f1.zip › Supp Material 4 - Custom-designed questionnaire.pdf]

Additional file 2 – Custom-designed questionnaire

柏金遜症非運動症狀評估量表 (Parkinson's Disease non-motor symptom assessment)

嚴重度 (severity): 0= 無此症狀; 1= 輕微; 2= 中度; 3= 嚴重; 4= 非常嚴重

頻率 (frequency): 1= 極少 (多於一星期而發); 2= 較少 (約一星期一發); 3= 頻發 (一星期多於一發); 4= 非常頻發 (約每天一發)

總積分計算 = 嚴重度 (severity) **x** 頻率 (frequency)

Severity: 0= absence of symptom; 1= mild; 2= moderate; 3= severe; 4= critical

Frequency: 1= rare (less than once in a week); 2= occasional (once in a week); 3= frequent (more than once in a week); 4= very frequent (almost daily)

症狀	嚴重度 (severity)	頻率 (frequency)	總積分
1. 打瞌睡：日常活動中，如在吃飯、看電視、看書的時候<br>Dozing: during daily activities e.g. mealtimes, watching television and read			
2. 疲乏：因精神狀態不佳（如疲倦，勞累）而不能參與一些日常活動<br>Fatigue: unable to participate in certain daily activities due to weariness			
3. 失眠：難以入睡或維持睡眠狀態 (如容易睡醒)<br>Insomnia: have difficulty in falling or staying asleep			
4. 眩暈：從臥位或坐位到站立時會感到頭暈，甚或站立不穩等不適<br>Vertigo: experience dizziness or loss of balance when standing up from a sitting or supine position			
5. 便秘：一星期少於三次<br>Constipation: defecate three times or less in a week			
6. 味覺異常或減弱<br>Impaired taste: loss of or distorted taste			
7. 流涎：不能自控或不自覺(清醒狀態時)<br>Involuntary or unconscious drooling			

8. 吞嚥困難：難以下嚥或進食<br>Dysphagia: Difficulty in swallowing or eating			
9. 尿頻：兩小時或以內排尿兩次或以上<br>Frequent urination: urinate twice or more in every two hours			
10. 夜尿：每晚睡覺時起床排尿兩次或以上<br>Nocturia: urinate twice or more every night			
11. 疼痛：無明顯成因的肢體或軀幹疼痛<br>Pain: experience pain in limbs or torso with no apparent cause			
